# *PredPsych*: A toolbox for predictive machine learning-based approach in experimental psychology research

**DOI:** 10.3758/s13428-017-0987-2

**Published:** 2017-12-12

**Authors:** Atesh Koul, Cristina Becchio, Andrea Cavallo

**Affiliations:** 1 Department of Psychology, University of Torino, Via Po, 14, 10123 Torino, Italy; 2 C’MoN, Cognition, Motion and Neuroscience Unit, Fondazione Istituto Italiano di Tecnologia, via Melen, 83, Genova, 1615 Italy

**Keywords:** Predictive approaches, Classification, Multivariate analysis, Clustering, Permutation testing

## Abstract

Recent years have seen an increased interest in machine learning-based predictive methods for analyzing quantitative behavioral data in experimental psychology. While these methods can achieve relatively greater sensitivity compared to conventional univariate techniques, they still lack an established and accessible implementation. The aim of current work was to build an open-source R toolbox – “*PredPsych*” – that could make these methods readily available to all psychologists. *PredPsych* is a user-friendly, R toolbox based on machine-learning predictive algorithms. In this paper, we present the framework of *PredPsych* via the analysis of a recently published multiple-subject motion capture dataset. In addition, we discuss examples of possible research questions that can be addressed with the machine-learning algorithms implemented in *PredPsych* and cannot be easily addressed with univariate statistical analysis. We anticipate that *PredPsych* will be of use to researchers with limited programming experience not only in the field of psychology, but also in that of clinical neuroscience, enabling computational assessment of putative bio-behavioral markers for both prognosis and diagnosis.

## Introduction

Experimental psychology strives to explain human behavior. This implies being able to explain underlying causal mechanisms of behavior as well as to predict future behavior (Kaplan, 1973; Shmueli, 2010; Yarkoni & Westfall, 2016). In practice, however, traditional methods in experimental psychology have mainly focused on testing causal explanations. It is only in recent years that research in psychology has come to emphasize prediction (Forster, 2002; Shmueli & Koppius, 2011). Within this predictive turn, machine learning-based predictive methods have rapidly emerged as viable means to predict future observations as accurately as possible, i.e., to minimize prediction error (Breiman, 2001b; Song, Mitnitski, Cox, & Rockwood, 2004).

The multivariate nature and focus on prediction error (rather than “goodness of fit”) confers these methods greater sensitivity and higher future predictive power compared to traditional methods. In experimental psychology, they are successfully used for predicting a variable of interest (e.g., experimental condition A vs. experimental condition B) from behavioral patterns of an individual engaged in a task or activity by minimizing prediction error. Current applications range from prediction of facial action recognition from facial micro-expressions to classification of intention from differences in the movement kinematics (e.g., Ansuini et al., 2015; Cavallo, Koul, Ansuini, Capozzi, & Becchio, 2016; Haynes et al., 2007; Srinivasan, Golomb, & Martinez, 2016). For example, they have been used to decode the intention in grasping an object (to pour vs. to drink) from subtle differences in patterns of hand movements (Cavallo et al., 2016). What is more, machine learning-based predictive models can be employed not only for group prediction (patients vs. controls), but also for individual prediction. Consequently, these models lend themselves as a potential diagnostic tool in clinical settings (Anzulewicz, Sobota, & Delafield-Butt, 2016; Hahn, Nierenberg, & Whitfield-Gabrieli, 2017; Huys, Maia, & Frank, 2016).

However, while the assets of predictive approaches are becoming well known, machine learning-based predictive methods still lack an established and easy-to-use software framework. Many existing implementations provide no or limited guidelines, consisting of small code snippets, or sets of packages. In addition, the use of existing packages often requires advanced programming expertise. To overcome these shortcomings, the main objective of the current paper was to build a user-friendly toolbox, “*PredPsych”*, endowed with multiple functionalities for multivariate analyses of quantitative behavioral data based on machine-learning models.

**Fig. 1:**
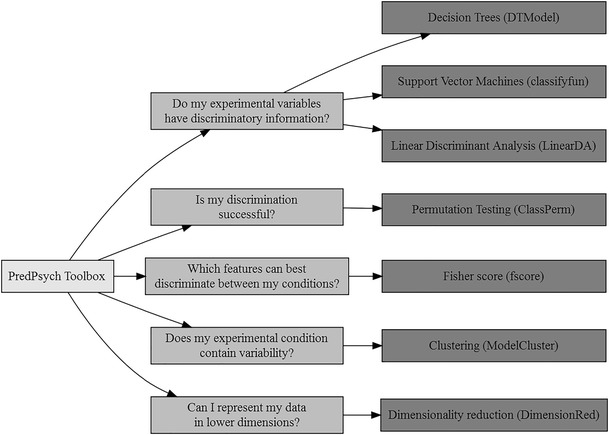
Overview of *PredPsych* functions. An overview of the research questions that can be addressed using *PredPsych* and the corresponding techniques

In the following, we present the framework of *PredPsych* via the analysis of a recently published multiple-subject motion capture dataset (Ansuini et al., 2015). First, we provide a brief description of the dataset and describe how to install and run *PredPsych*. Next, we discuss five research questions that can be addressed with the machine learning framework implemented in *PredPsych*. We provide guided illustrations on how to address these research questions using *PredPsych* along with guidelines for the best techniques to use (for an overview see Fig. 1) and reasons for caution. Because the assets of predictive approaches have been recently discussed elsewhere (Breiman, 2001b; Shmueli, 2010), we only briefly deal with them here.

## Data description

**Fig. 2:**
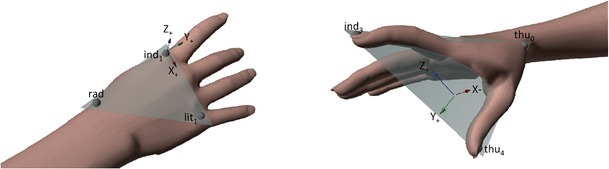
Hand model for estimating kinematics variables. Schematic showing the hand model depicting global and local frames of reference used for the calculation of kinematics variables

The data utilized for the current paper employ part of the motion capture dataset freely available in the publication (Ansuini et al., 2015). This dataset was obtained by recording 15 naïve participants performing reach-to-grasp movements towards two differently sized objects: a small object (i.e., hazelnut) and a large object (i.e., grapefruit). Movements were recorded using a near-infrared camera motion capture system (frame rate 100 Hz; Vicon System). Each participant was equipped with lightweight retro-reflective hemispheric markers placed on the radial aspect of the wrist, the metacarpal joint and the tip of the index finger, the metacarpal joint of the little finger, the trapezium bone of the thumb, and the tip of the thumb (Fig. 2). Subsequently, kinematic features of interest were estimated based on global frame of reference of motion capture system (F-global) and a local frame centered on the hand (F-local) (Fig. 2):

- Wrist Velocity, defined as the module of the velocity of the wrist marker (mm/sec);
- Wrist Height, defined as the z-component of the wrist marker (mm);
- Grip Aperture, defined as the distance between the marker placed on thumb tip and that placed on the tip of the index finger (mm);
- x-, y-, and z-thumb, defined as x-, y-, and z-coordinates for the thumb with respect to F-local (mm);
- x-, y-, and z-index, defined as x-, y-, and z-coordinates for the index finger with respect to F-local (mm);
- x-, y-, and z-finger plane, defined as x-, y-, and z-components of the thumb-index plane, i.e., the three-dimensional components of the vector that is orthogonal to the plane. This plane is defined as passing through thu0, ind3, and thu4, with components varying between +1 and ˗1.

All kinematic variables were expressed with respect to normalized movement duration (from 10 % to 100 %, at increments of 10 %; for detailed methods, please refer to (Ansuini et al., 2015). The dataset in the toolbox consists of a 848 × 121 matrix, where variables are arranged in columns (the first column represents the size of the grasped object, 1 = “small” object; 2 = “large” object; the other columns represent the kinematic variables) and observations (n = 848) are present in rows.

## Toolbox installation and setup

To install the toolbox, the user has first to install the programming language R (R Core Team (2016) www.r-project.org). For easier use of R tools, we recommend using the interface *RStudio* (https://www.rstudio.com/). After successful installation of R environment, the command *install.packages(‘PredPsych’,dependencies=TRUE)* can be used to install the package (in case you are prompted to select a Comprehensive R Archive Network (CRAN) repository, choose the one located closest to you). All the packages required for the installation of the package will be installed automatically. The package can then be loaded with the command *library(PredPsych)*. This command loads all the functions as well as the data from the experiment.

## Research questions

In the current paper, we discuss the following five research questions and illustrate their implementation in *PredPsych*:

1. Do my experimental conditions have discriminatory information?
2. Is my discrimination significant?
3. Which features/variables can best discriminate between my conditions?
4. Do my experimental conditions contain variability?
5. Can I represent my data in lower dimensions?

### Q1. Do my experimental variables have discriminatory information?

This kind of question arises when researchers are interested in understanding whether properties of the collected data (i.e., data features) encode enough information to discriminate between two or more experimental conditions (i.e., classes or groups). This goes beyond asking whether the data features are significantly different among the classes; it also requires to determine whether and to what extent data features can be combined to reliably predict classes and, when errors are made, what is the nature of such errors, i.e., which conditions are more likely to be confused with each other (Tabachnick & Fidell, 2012).

Questions of this sort are perfectly suited for a classification analysis (Bishop, 2006; Hastie, Tibshirani, & Friedman, 2009). Classification analysis is a supervised machine learning approach that attempts to identify holistic patters in the data and assigns classes to it (classification). Given a set of features, a classification analysis automatically learns intrinsic patterns in the data to predict respective classes. If the data features are informative about the classes, a high classification score is achieved. Such an analysis thus provides a measure about whether the data features “as a whole” (i.e., in their multivariate organization) contain discriminatory information about the classes. Currently, *PredPsych* implements three of the most commonly used algorithms for classification: Linear Discriminant Analysis, Support Vector Machines and Decision Tree models (see Appendix 1 for guidelines on classifier selection).

#### Linear discriminant analysis (LDA)

The simplest algorithm for classification based analysis is the Linear Discriminant Analysis (LDA). LDA builds a model composed of a number of discriminant functions based on linear combinations of data features that provide the best discrimination between two or more classes. The aim of LDA is thus to combine the data feature scores in a way that a single new composite variable, the discriminant function, is produced (for details see Fisher, 1936; Rao, 1948). LDA is closely related to logistic regression analysis, which also attempts to express one dependent variable as a linear combination of other features. Compared to logistic regression, the advantage of LDA is that it can be used also when there are more than two classes. Importantly, LDA should be used only when the data features are continuous.

#### Implementation in PredPsych

LDA is implemented in *PredPsych* as *LinearDA* function in the toolbox and utilizes the *mass* package (Venables & Ripley, 2002). This function mandatorily requires inputs in the form of a dataframe^1^ (*Data*) and a column for the experimental conditions^2^ (*classCol*).^2^ Optionally, if the researcher would like to perform classification analysis only for a subset of possible features, he/she can also select only specific columns from the dataframe (*selectedCols*).

Additional optional inputs control the type of cross-validation to be performed (Appendix 2): *cvType* = “folds” for k-fold cross-validation, *cvType* = “LOSO” for leave-one-subject-out procedure, *cvType* = “LOTO” for leave-one-trial-out procedure, *cvType* = “holdout,” for partition based procedure. If no input is provided for this parameter, then *LinearDA* function performs a k-fold cross-validation^3^ splitting the dataset into 10 folds and repeatedly retaining one fold for testing the model and utilizes the other folds for training the model (for details on all other parameters that can be set for the *LinearDA* function, see *PredPsych* manual).

By default, the *LinearDA* function outputs the accuracy of the classification analysis and prints the confusion matrix of the actual and the predicted class memberships for the test data. However, the researcher can also optionally choose to output extended results (parameter: *extendedResults =* TRUE), including the LDA model, accuracy as well as confusion matrix metrics (see Appendix 3).

As an illustrative example, we can select the kinematic features for the first time interval (at 10 % of the movement) as data features and the first column (object size) as class. We set the cross-validation type as “holdout” and use 80 % of the data for training and the remaining 20 % of the data for testing (*cvType* = “holdout”). We generate only the accuracy as output. Alternatively, setting *extendedResults* to TRUE, we can also obtain the LDA model. We observe that the LDA model obtains an accuracy of 57 % on this dataset, successfully predicting 51/83 cases for the “small” (1) class and 45/85 cases for the “large” (2) class in the test dataset (Table 1).
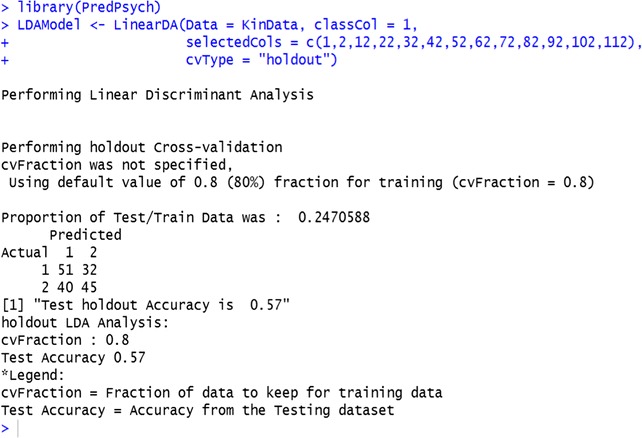

**Table 1:** Confusion matrix generated by LDA. Rows represent the actual class of the data while the columns represent the predicted class membership

	Predicted 1	Predicted 2
Actual 1	51	32
Actual 2	40	45

The model obtained can then be used to predict new dataset – a new set of data that has never been used for training or testing the model (e.g., data to be collected in follow-up experiments). This can be accomplished using the same function *LinearDA* using the parameters *extendedResults* = TRUE and inputting the new data features using the parameter *NewData*. The predicted class membership for each case of new data is stored in the LDAModel variable (visible using the command – LDAModel$fitLDA$newDataprediction).

#### Support vector machines (SVMs)

More sophisticated algorithms like Support Vector Machines (SVMs) can be also applied to test whether the data features obtained from an experiment encode sufficient discriminatory information between conditions. Similarly to LDA, SVMs try to discriminate between classes/conditions. However, instead of finding a linear function that separates the data classes, SVMs try to find the function that is farthest from data points of any class (Cortes & Vapnik, 1995; Duda, Hart, & Stork, 2000; Vapnik, 1995). This leads to an optimal function that best separates the data classes. Since the data classes may not necessarily be linearly separable (by a single line in 2D or a plane in 3D), SVMs use a kernel function^4^ to project the data points into higher dimensional space. SVMs then construct a linear function in this higher dimension. Some of the commonly used kernel functions are linear, polynomial and radial basis function.

#### Implementation in PredPsych

Classification using SVMs is implemented as a classification function named *classifyFun* and utilizes the package *e1071* (Meyer et al., 2017). This function additionally tunes parameters (searches for optimal parameter values) for one of commonly used kernel function – radial basis function (RBF). RBF kernel requires two parameters: a cost function C and a Gaussian kernel parameter gamma. The procedure implemented in *PredPsych* performs cross-validation and returns tuned parameters (based on a separate division of the data). To obtain tuned parameters, the input dataset is divided into three parts. These three dataset divisions are used for tuning parameters, training and testing without reusing the same data. If, however, the tuning option is not selected, the data is divided only in training and testing parts. These divisions ensure avoiding biases in the classification analysis.

For illustrative purposes, we submit the same data and the same features used in LDA (kinematic features at 10 % of the movement) to SVMs. Similar to the function *LinearDA*, *classifyFun* requires the dataframe (*Data*) and a column for the experimental conditions (*classCol*) as inputs. Additionally, other inputs can be provided indicating the following: the type of cross-validation to be performed (*cvType* = “holdout”, “folds”, “LOTO”, or “LOSO”), subset of features to be selected (*selectedCols*): a logical parameter (TRUE or FALSE) that states whether to find optimal SVM parameters (*tune = TRUE*) or not (*tune = FALSE*), the parameter that specifies ranges in which to search for optimal SVM parameters of gamma and cost (*ranges*), a cost function parameter (*C*) and a radial basis kernel parameter (*gamma*) (see *PredPsych* manual for other parameters that can be set). As a default, the function uses radial basis function (radial) as the kernel and performs a 10-fold cross-validation. As in LDA, here we used the same data and “holdout” cross-validation scheme. A test accuracy of 65 % is obtained.
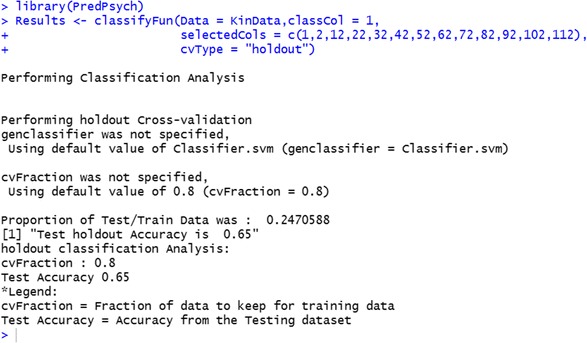


As for the LDA, the SVM model obtained can be used to make predictions about the class/condition of a new dataset using the parameter setting of *extendedResults* = TRUE and inputting new data features in *NewData*. Results of the analysis will be available in the variable Results (as Results$classificationResults$newDataprediction).

#### Decision tree models

Another class of algorithms that a researcher can employ to predict the outcome from the data are Decision tree (DT) models (Loh, 2011). DT models fall under the general “Tree-based methods” involving generation of a recursive binary tree (Hastie et al., 2009). In terms of input, DT models can handle both continuous and categorical variables as well as missing data. From the input data, DT models build a set of logical “if …then” rules that permit accurate prediction of the input cases.

DT models are especially attractive types of models for two reasons. First, they are more flexible than regression methods and, unlike linear regression methods, can model nonlinear interactions. Second, they provide an intuitive representation based on partitioning – which variables combined in which configuration can predict the outcome (Breiman, Friedman, Stone, & Olshen, 1984). DT models implemented in the *PredPsych* toolbox are Classification and Regression Tree (Breiman et al., 1984), Conditional Inference (Hothorn, Hornik, & Zeileis, 2006), and Random Forest (Breiman, 2001a).

#### Implementation in PredPsych

DT models in *PredPsych* are implemented as function *DTModel* employing the *rpart* package (Therneau et al., 2015). This function takes as mandatory inputs a dataframe (*Data*), a column for the experimental conditions (*classCol*), and the type of DT model to use (*tree*): *tree = “CART”,* for a full CaRT model, *tree = “CARTNACV”,* for a CaRT model with cross-validation (removing the missing values), *tree = “CARTCV”,* for a CaRT model with cross-validation (the missing values being handled by the function *rpart*), *tree = “CF”,* for Conditional Inference, *tree = “RF”,* for Random Forest. The function *rpart* handles the missing data by creating surrogate variables instead of removing them entirely (Therneau, & Atkinson, 1997). This could be useful in case the data contains a higher number of missing values.

Additional optional arguments that can be provided are the subset of data features (*selectedFeatures*), type of cross-validation (*cvType* = “holdout,” “folds,” “LOTO,” or “LOSO”) and related parameters for cross-validation (see *PredPsych* manual for further details on other parameters that can be set). The output of this operation returns a decision tree and, if appropriate, accuracy results and a figure from the chosen DT model. In cases of CART, the tree is automatically pruned using a value of complexity parameter that minimizes the cross-validation accuracy in the training dataset. The resulting figures thus display the pruned tree.

**Fig. 3:**
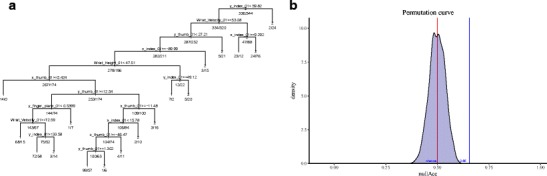
Results from decision trees and permutation testing. (**a**) Classification and regression tree for classification of movements directed towards a small (1) vs. a large (2) object. (**b**) A null distribution density profile depicting significant permutation results for classification of movement towards a small vs. a large object

As an illustrative example, we use the function *DTModel* to generate a CART model using the same kinematics data as in the previous examples (using features at 10 % of the movement). The resulting feature tree using *tree = “CARTCV”* showing “if…then” rules is depicted in Fig. 3a. The results indicate that if the index finger deviates more than 59.82 mm on the y-coordinate, then the movement is directed towards the large object 24 out of 26 times (92.30 %). Alternatively, if the y-coordinate of the index finger is less than 59.82 mm and wrist velocity is greater than 53.08 mm/s, then the movement is directed towards the small object in 287 out of 519 (55.30 %) cases (Fig. 3a). The corresponding cross-validation test accuracy obtained by utilizing 80 % of the entire dataset for training is 62 %.
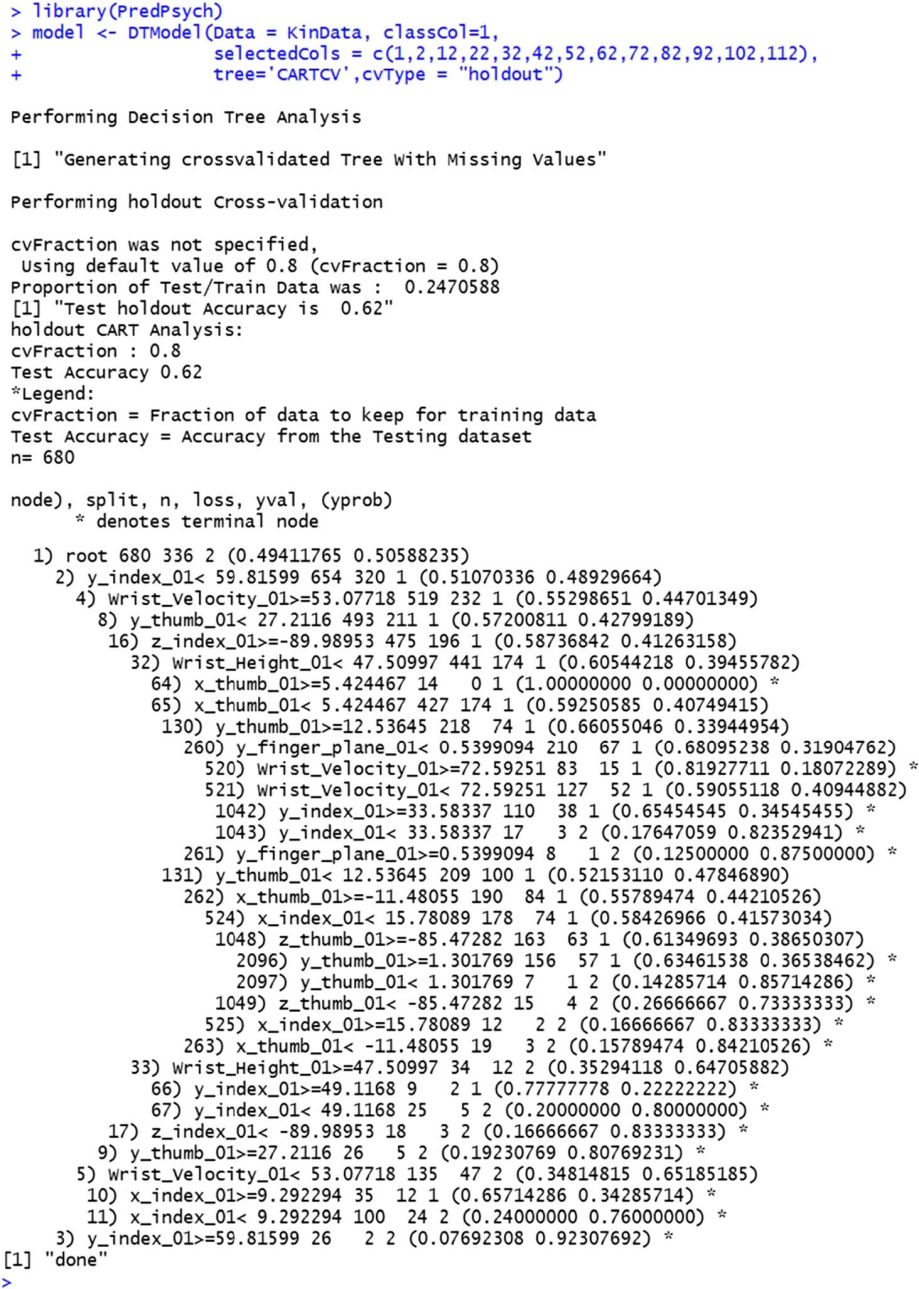

Further, the obtained DT model can then be used to make predictions about classes/conditions of a new dataset by setting the parameters – *extendedResults* = TRUE and inputting new data features as *NewData*. The Results for the new dataset would be available in model variable as model$fit$newDataprediction.

### Q2. Is my discrimination successful?

Question 1 informs a researcher on the extent of discriminatory power of the variables collected in an experiment, but it does not comment on the statistical significance of the discrimination. For this reason, after obtaining classification results, a researcher might ask if the results obtained reflect a real class structure in the data, i.e., whether they are statistically significant. This is especially important when the data, as in most psychological research, have a high dimensional nature with a low number of observations. In such cases, even if the classification algorithm produces a low error rate, it could be that classification does not reflect interdependencies between the data features for classification, but rather differences in value distributions inside the classes (Ojala & Garriga, 2010). The data themselves, however, may have no structure. One way to assess whether the classifier is using a real dependency in the data is to utilize a permutation based testing (Ojala & Garriga, 2010). Permutation tests are a set of non-parametric methods for hypothesis testing without assuming a particular distribution (Good, 2005). In case of classification analysis, this requires shuffling the labels of the dataset (i.e., randomly shuffling classes/conditions between observations) and calculating the accuracies obtained. This process is repeated a number of times (usually 1,000 or more times). The distribution of accuracies is then compared to the actual accuracy obtained without shuffling. A measure of how many times accuracies obtained by randomization are higher than the actual accuracy provides information about significance of the classification. That is, the percentage of cases where randomly shuffled labels give accuracies higher than actual accuracy corresponds to an estimate of the p-value. P-values are calculated either using exact or approximate procedure depending on the number of possible permutations (Phipson & Smyth, 2010). Given an alpha level, the estimated p-value provides information about the statistical significance of the classification analysis.

#### Implementation in PredPsych

Permutation testing in *PredPsych* is implemented as *ClassPerm*. The main inputs necessary for the function are the dataframe (*Data*) for classification and a column for the experimental conditions (*classCol*). Optionally, a classifier function (*classifierFun*) can be provided as an input to the permutation function. This function can be any function that returns mean accuracy of classification (e.g., *LinearDA*). A specific number of simulations (*nSims*) can also be input as an optional input to the function. If no *classifierFun* is provided, a default SVM classifier with k-fold cross-validation is utilized. The number of simulations defaults to 1,000 if no input is provided. The function, in addition to calculating p-value for the classification, also generates a figure for representation of the null distribution and classification accuracy (with chance level accuracy as red vertical line and actual classification accuracy with a vertical blue line) (Fig. 3b).

We utilize the same data and holdout cross-validation as in previous classification analyses to verify if the classification we obtained is significant or not. Our results suggest a p-value of 0.001. As the p-value is lower than the alpha value of 0.05 commonly used in psychology research, this suggests that the classification accuracy obtained is significant.
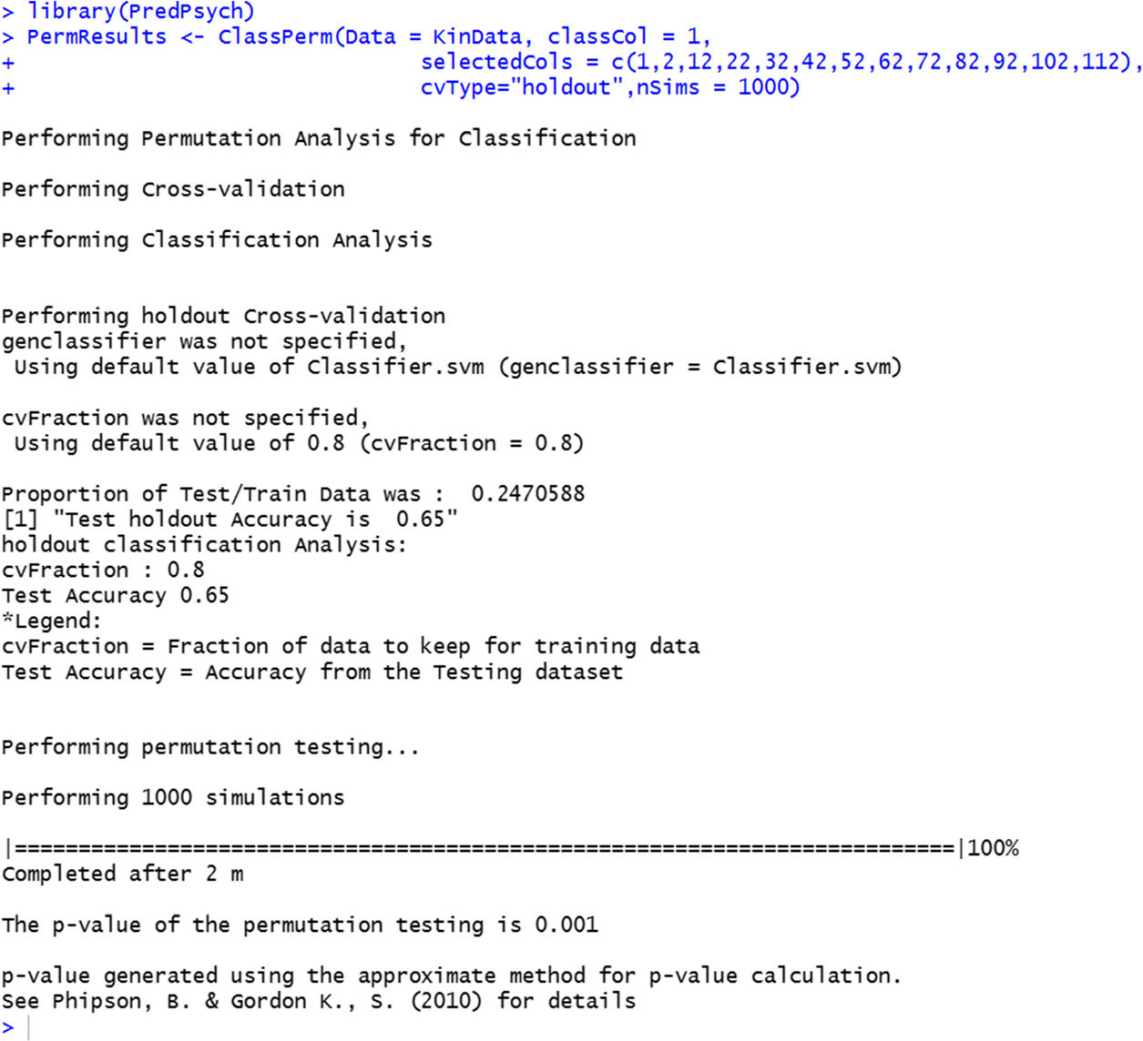


### Q3. Which features/variables can best discriminate between the conditions?

Classification analysis provides information about whether data features contain discriminatory information. However, there are cases in which hundreds of features are used as inputs for the classification and many of them might not contribute (or not contribute equally) to the classification. This is because while certain features might favor discrimination, others might contain mere noise and hinder the classification (i.e., increase the prediction error). In such a case, it is advisable to perform some sort of feature selection to identify the features that are most important for a given analysis. In a first screening, the researcher can remove problematic features based on a set of criteria (e.g., percentage of missing values). Then, a rank can be assigned to the remaining features based on their importance. As a third step, according to their rank, the features that aid classification can be retained while those that merely add noise to the classification can be eliminated. Prediction errors can, thus, be evaluated on this subset of features instead of using all the features present.

Feature selection has been one of the actively debated topics in machine learning (Chen & Lin, 2006; Raftery & Dean, 2006; Saeys, Inza, & Larrañaga, 2007), especially since a good selection can indeed help boost or fine-tune the classification. One of the measures commonly used for feature selection is the Fisher score (F-score) (Chen & Lin, 2006; Duda et al., 2000). F-score provides a measure of how well a single feature at a time can discriminate between different classes. The higher the F-score, the better the discriminatory power of that feature. Mathematically, the F-scores represent the ratio between the discrimination between the classes and the discrimination within the classes i.e., the ratio between-class scatter to within-class scatter as given by the following formula:

$$ F=\frac{\parallel {\overrightarrow{\mu}}^P-{\overrightarrow{\mu}}^Q{\parallel}_2^2}{tr\left({\varSigma}^P\right)+ tr\left({\varSigma}^Q\right)} $$

Where ${\overrightarrow{\mu}}^P$ and ${\overrightarrow{\mu}}^Q$ are means of the feature vector and ∑^*P*^ and ∑^*Q*^ are the covariance matrices for *P* and *Q* classes respectively, *tr*() denotes trace of a matrix and ‖⋅‖_2_ denotes the Euclidean norm.

Even though this approach has the limitation of calculating scores independently for each feature, the measure is easy to calculate. An alternate approach for calculating importance of features is using the feature importance scores from random forest trees (also implemented using the DTModel function with tree parameter as “RF”).

#### Implementation in PredPsych

F-scores are implemented using the function *fscore* in *PredPsych*. The function requires a dataframe (*Data*) as an input and a column for the experimental conditions (*class*Co*l*). Additionally, it requires feature columns (*featureCol*) for which the scores have to be calculated. For ease of understanding, the function outputs a named numeric structure with names of the features and their corresponding F-scores. We utilize the features used in previous analyses to calculate their discriminatory power individually. We observe that the discriminatory power at 10 % of the movement is highest for Wrist Velocity (0.055) followed by Grip Aperture (0.030) and y-index (0.012). Features such as Wrist Height, x-index, z-index, z-thumb, x-finger plane, and y-finger plane do not contribute to any discriminatory power at 10 % of the movement (Table 2).
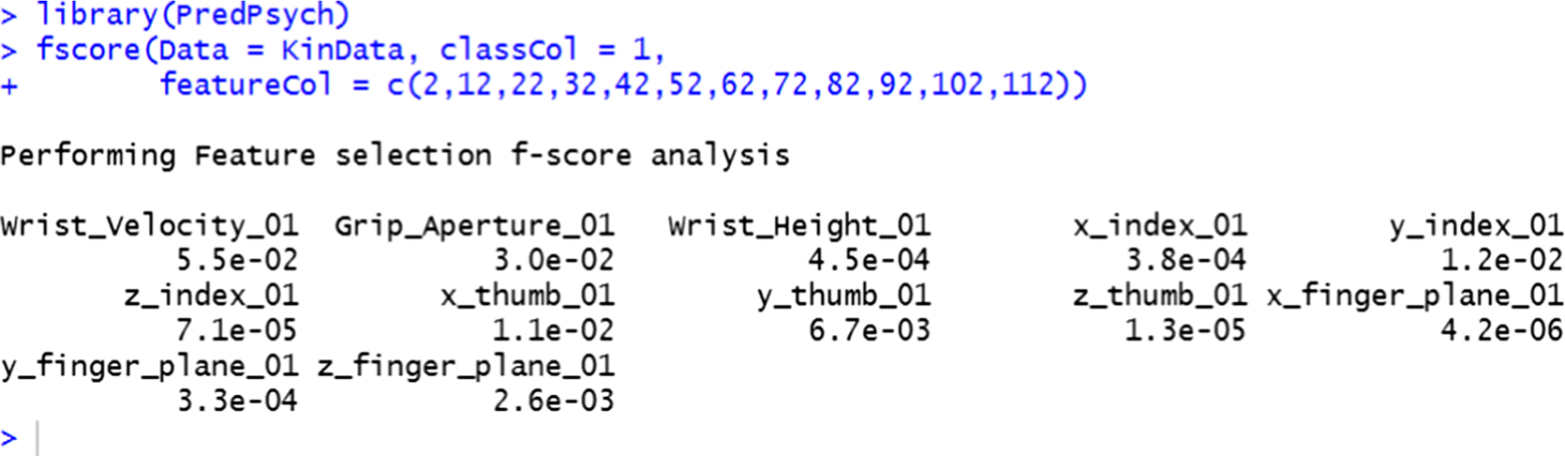

**Table 2:** Feature selection results. F-scores for all the features at 10 % of the movements towards small vs. large object

Data features	F_scores
Wrist Velocity 01	0.055
Grip Aperture 01	0.030
Wrist Height 01	0.00045
x_index 01	0.00038
y_index 01	0.012
z_index 01	7.10e-05
x_thumb 01	0.011
y_thumb 01	0.0067
z_thumb 01	1.30E-05
x_finger plane 01	4.20e-06
y_finger plane 01	0.00033
z_finger plane 01	0.0026

### Q4. Does my experimental conditions contain variability?

Variability in data has long been considered as unwanted noise arising from inherent noise in sensory or motor processing (Churchland, Afshar, & Shenoy, 2006; Jones, Hamilton, & Wolpert, 2002). More recent studies, however, suggest that this variability might reflect slight differences in the underlying processes, especially individual-based differences (Calabrese, Norris, Wenning, & Wright, 2011; Koul, Cavallo, Ansuini, & Becchio, 2016; Ting et al., 2015). Consequently, many researchers are attempting to gain a better understanding of their results in terms of intrinsic variability of the data. When the source of this variability is not clear, researchers have to rely on exploratory approaches such as clustering or non-negative factorization.

Clustering approaches partition data features in subsets or clusters based on data similarity. Each cluster comprises observations that are similar to each other compared to those in the other clusters (for an overview see Han, Kamber, & Pei, 2012). Unlike classification analyses, clustering analysis does not require class labels but utilizes the data features to predict subsets and is thus an unsupervised learning approach.

Clustering has previously been utilized for a number of applications in data sciences ranging from image pattern recognition, consumer preferences, and gene expression data to clinical applications. All clustering approaches need the specification of a specific cluster number in addition to the data features. In most of the cases (unless there is an *a-priori* information), this number of clusters is chosen arbitrarily. Model based clustering approaches provide a methodology for determining the number of clusters (Fraley & Raftery, 1998). In a model based approach, data are considered to be generated from a set of Gaussian distributions (components or clusters), i.e., as a mixture of these components (mixture models). Instead of using heuristics, model based clustering approximates Bayes factor (utilizing Bayesian Information Criterion) to determine the model with the highest evidence (as provided by the data). The generated model from this approach, in contrast to other clustering approaches, can further be used to predict new data classes from data features.

#### Implementation in PredPsych

Clustering analysis is implemented in *PredPsych* as *ModelCluster*. This function performs model based clustering using *mclust* package (Fraley & Raftery, 2007). *ModelCluster* requires a dataframe (*Data*) as mandatory input. Optionally, it can be utilized to predict class memberships for a new set of data utilizing the model just obtained (*NewData*). Other optional arguments include number of components for which BIC has to be calculated (*G*). For the implementation, we utilize the full KinData dataset to examine presence of regions with varying motor variability in a motor act. We calculate optimal number of clusters at each time interval (from 10 % of the movement to 100 %).
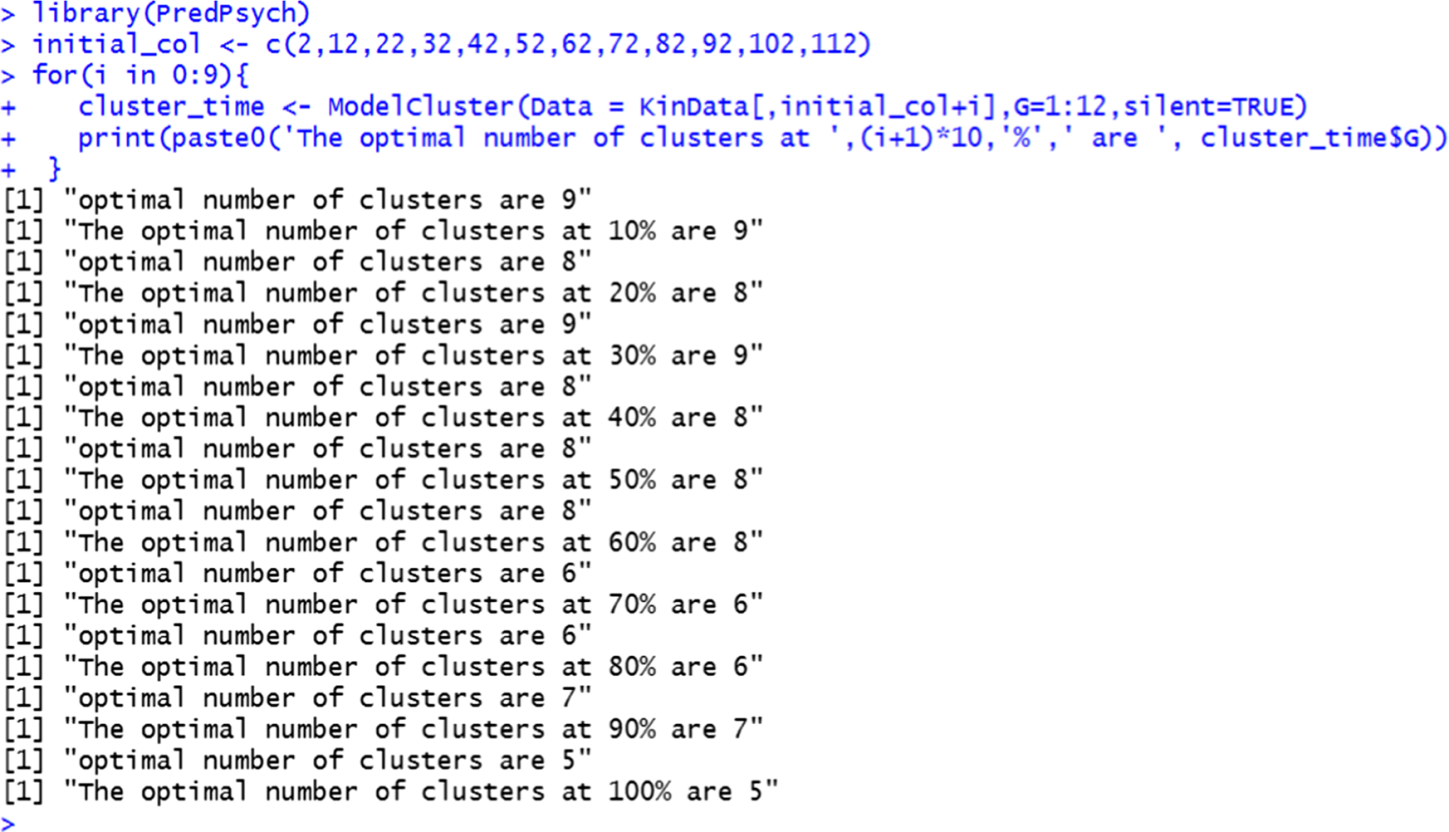


We obtain that the number of clusters reduces as the movement progresses starting from nine clusters (at 10 %) to five clusters (at 100 % of the movement). This is in agreement with recent propositions that biological constraints and affordances shape so-called “don’t care” or “bottle-neck” regions (Beer, Chiel, & Gallagher, 1999; Ting et al., 2015). These regions correspond to high and low motor variability, respectively.

### Q5. Can I represent my data in lower dimensions?

While the excitement surrounding multivariate analyses of quantitative behavioral data is still growing, researchers have also come to realize that the nature and volume of multivariate data pose severe challenges for making psychological sense of these data. Variables in such data often are correlated with each other making the interpretation of the effects difficult. In addition, high-dimensionality can have adverse effects on classification analyses. Problems of over-fitting (i.e., classification model exhibiting small prediction error in the training data but much larger generalization error in unseen future data), in particular, can occur when the number of observed variables is higher than the number of available training sample.

To escape the curse of dimensionality (Bellman, 1957), it is sometimes imperative to construct interpretable low-dimensional summaries of high-dimensional data. Dimensionality reduction has been proven useful for generating relatively independent data features, obtaining higher and more generalizable classification results (lower prediction errors), and aiding the interpretability of the results. Various models have been developed for such dimensionality reduction, including Principal Component Analysis (PCA), Independent Component Analysis (ICA), Non-negative matrix factorization (NMF), Multidimensional scaling (MDS) etc. *PredPsych* currently implements two of the most commonly used models – MDS and PCA.

MDS, similarly to other mentioned techniques, attempts to project the multidimensional data into lower dimensions (Bishop, 2006; Cox & Cox, 2000). In contrast to PCA, MDS tries to preserve the original distance relationship present in the multidimensional space for projections in the lower dimension. PCA on the other hand, attempts to preserve the original co-variance between the data points.

#### Implementation in PredPsych

Dimensionality Reduction in *PredPsych* is implemented as the function *DimensionRed*. This function as mandatory inputs requires the dataframe (*Data*) and the selected columns (*selectedCols*) for which the dimensionality has to be reduced. Additional inputs can be provided for visualizing the first two reduced dimensions – *outcome* (class of the observation present as rows of the dataframe) and *plot* (a logical indicating if the plot should be displayed).

To provide an illustration, we display reduced dimensions for two kinematic parameters – Wrist Velocity and Grip Aperture - from 10 % to 100 % of movement duration (10 time points). For each kinematic feature, we reduced the dimension from 10 to 2.
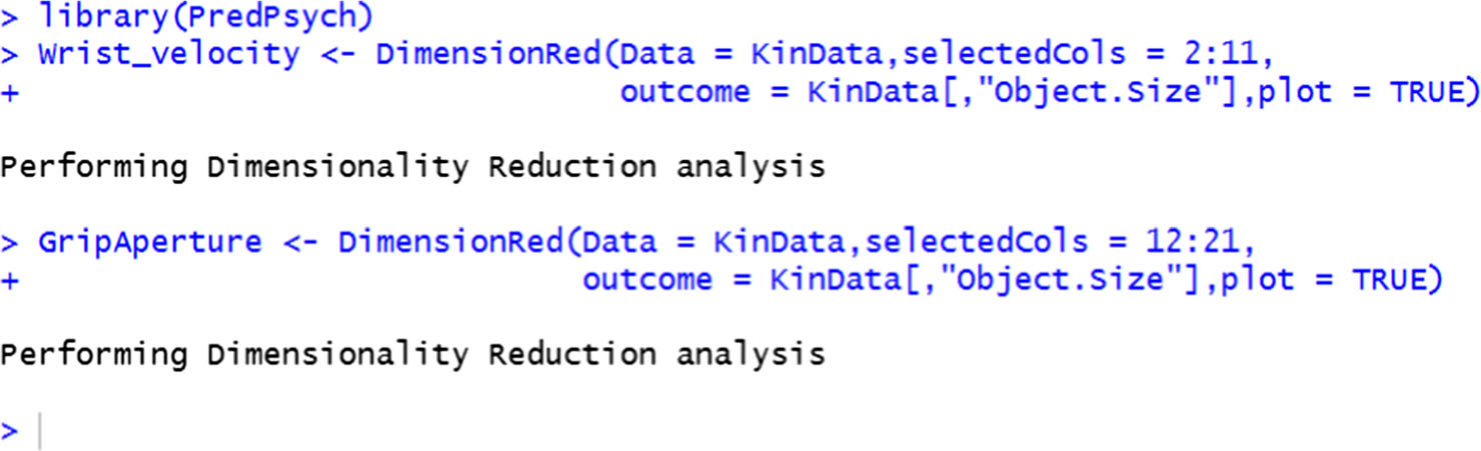


**Fig. 4:**
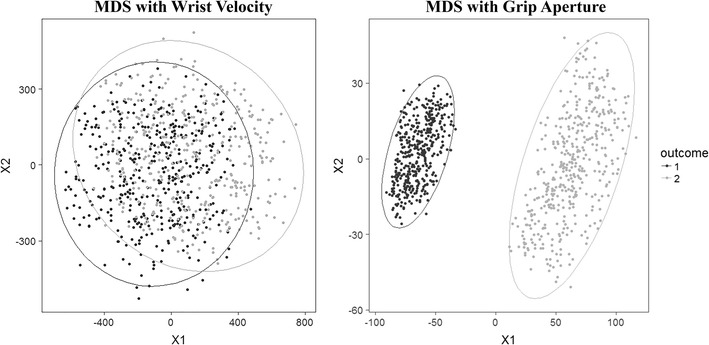
Dimensionality Reduction results. A higher separation is found between small and large object for Grip Aperture compared to Wrist Velocity in the reduced 2D space

The results from this analysis suggest a higher separation between ‘small’ and ‘large’ object for Grip Aperture compared to Wrist Velocity (Fig. 4).

## Discussion and conclusions

Causal explanatory analyses in experimental psychology have been recently complemented by predictive methods based on machine-learning models. These methods allow an increased sensitivity and greater predictive power compared to traditional explanatory approaches. Resources available to researchers for their implementations, however, are still surprisingly scarce. Without a proper framework, utilizing these analyses requires substantial expertise and is frequently opaque to the non-experts.

*PredPsych* aims at providing a comprehensive and user-friendly software framework for the use of predictive methods based on machine learning in experimental psychology. With this framework, we present *PredPsych* by outlining the type of questions that can be answered using the functions implemented in the package. Furthermore, we provide examples on how to apply these functions and offer suggestions on the choice of the parameters.

Navigating by trial-and-error is often the default approach in machine learning. *PredPsych*, instead, encourages researchers to formulate their research questions first and, then, based on the specific question, select the most appropriate technique. A distinctive feature of *PredPsych* in comparison to other available packages is its tailoring to experimental psychology. This is both a strength and limitation: a strength, in that it makes the application of the implemented functions accessible to experimental psychologists with limited programming experience; a limitation, in that the resulting framework is less abstract and thus less reusable in other contexts. Other packages, such as Scikit-learn, for example, implement generic functions usable in various domains, ranging from spam detection and image recognition to drug response and stock prices. These packages are thus more flexible but also more difficult to use, as their adaptation requires the programming of specific scripts.

We anticipate that *PredPsych* along with the illustrations provided in this paper will favor the spread of predictive approaches across various sub-domains of experimental psychology. Moreover, we hope that the framework of *PredPsych* will be inspiring and informative for the clinical psychology community, enabling clinicians to ask new questions – questions that cannot be easily investigated using traditional statistical tools. Overall, machine learning-based predictive methods promise many opportunities to study human behavior and develop new clinical tools.

## Appendix 1: Classifier selection

The choice of which specific classifier to use is an actively researched area of study (Douglas, Harris, Yuille, & Cohen, 2011; Kiang, 2003; Kim, 2009; Song, Mitnitski, Cox, & Rockwood, 2004). Inherently, no classification method is superior to the other and in most cases, the choice depends on multiple factors like classifier assumptions, sample size, model training speed, model complexity, result interpretability, and parameter settings, among others (Duda, Hart, & Stork, 2000). As a rule of thumb, simpler (linear, fewer features) classifiers are preferred over complex classifiers in order to avoid overfitting and for generalisation purposes. To aid the selection of a classifier, we highlight below certain properties of the three classifiers described in the current study (Table 3):

**Table 3:** Guidelines and properties for three classifiers implemented in PredPsych.

	Classifier type	Data type/ assumption	Computational cost/complexity	Output	Interpretability
Linear Discriminant Analysis	Linear	Preferably normality assumption, identical covariance matrices	Simple, lower computation time	Prediction error, discriminant scores for features	Easy
SVM	Linear, non-linear	No specific data distribution	Higher complexity, higher time consumption	Prediction error	Can be difficult to interpret
Decision Tree Models	Linear, non-linear	Can handle nominal data, no specific data distribution	Simple, rapid classification	Prediction error, if else rules	Easy

## Appendix 2: Cross-validation

### Definitions

Cross-validation refers to a set of techniques for partitioning data into complementary sets for obtaining objective, independent, and unbiased estimate of learned model performance (Browne, 2000; Han, Kamber, & Pei, 2012; Kohavi, 1995). Using cross-validation, one splits the data into training and testing datasets. The model is first trained on the training dataset and the model is subsequently evaluated on data from the test dataset (data that the model hasn’t encountered before). This splitting of the datasets could be performed in multiple different ways. Some of the common (but not exhaustive) ways to perform the cross-validation are as follows:

1. k-fold cross-validation
2. Leave-one-subject-out cross-validation
3. Leave-one-trial-out cross-validation
4. Holdout cross-validation.
5. k-fold cross-validation

k-fold cross-validation involves splitting the dataset into a certain number of equally sized folds (k). Of these folds, one fold is retained as testing dataset while the others (k-1) are used for the training purposes. This procedure is repeated k-times, each time selecting a different fold for testing purposes and the other folds (k-1) as training dataset. Consequently, k different accuracies are produced from the procedure. A value of k = 10 is generally used as a rule of thumb for the number of folds.

2. Leave-one-subject-out cross-validation

Leave-one-subject-out approach, similar to k-fold cross-validation, splits the dataset multiple times into training and testing. However, instead of creating k-folds, this procedure splits the dataset according to the number of subjects in the dataset. One subject is selected for the testing purposes while the other subjects are used for the training the model. This procedure is repeated until all the subjects have been used as test dataset.

3. Leave-one-trial-out cross-validation

Leave-one-trial-out approach, in contrast to leave-one-subject-out approach, splits the dataset based on the number of dataset samples. That is, one sample from the dataset is retained for testing and the rest of the samples are used to generate the model. This sampling is repeated until all the samples have been used as a testing data point. Leave-one-trial-out procedure can also be visualized as a k-fold cross-validation procedure with the k being equal to the number of samples in the dataset.

4. Holdout cross-validation

Holdout is the simplest kind of cross-validation, often considered as a validation method, because contrary to previous methods, it performs the test/train split only once. A portion of the data is randomly selected as test dataset while the rest of the data is utilized as training dataset. A fraction of 1/3 is generally used as test data for holdout procedures.

### Selection of a scheme

The selection of a specific cross-validation procedure depends on multiple factors related to the sample size, experimental design among many others and is still an actively researched field (Borra & Di Ciaccio, 2010; Gong, 1986; Kim, 2009; Varoquaux et al., 2017). As a general guideline, a 10-fold cross-validation procedure is recommended.

## Appendix 3: Alternate measures of classifier performance

### Definitions

The simplest measure of evaluating the performance of a classifier is the classifier accuracy, i.e., classification rate.

$$ Accuracy=\frac{no. ofcasesidentifiedcorrectly}{no. oftotalcases} $$

However, such a measure only provides an overall information about the classifier performance. In order to obtain a detailed analysis of the performance of the classifier, confusion matrix analysis can be employed. A confusion matrix is simply a summary table comprised of frequencies of predictions made by the classifier for each possible class.

**Table 4:** Confusion matrix for a two-class classification analysis. All four possible outcomes are demonstrated

	Predictions		
Actual		Class 1	Class 2
	Class 1	True Positive	False Negative
	Class 2	False Positive	True Negative

A confusion matrix is indispensible for dissecting the classifier performance especially in cases with imbalanced class distributions as well as in multiclass classifications. Consider a case where one of the classes is overrepresented and the classifier achieves an overall accuracy of 80 %. The classifier can achieve this classification by simply making correct predictions for the class with higher number of cases while consistently getting the other class wrong.

There are multiple metrics that can be extracted from the confusion matrix that reflect multiple properties of the classifier. Some of the metrics and their associated definitions are provided below:

$$ {\displaystyle \begin{array}{l} Sensitivity=\frac{TP}{\left( TP+ FN\right)}\\ {} Specificity=\frac{TN}{\left( TN+ FP\right)}\\ {} Precision=\frac{TP}{\left( TP+ FP\right)}\\ {} Recall=\frac{TP}{\left( TP+ FN\right)}\\ {}{F}_1=2\ast \frac{\left( Precision\ast Recall\right)}{\left( Precision+ Recall\right)}\end{array}} $$

### Implementation in PredPsych

All the functions for classification analysis (LinearDA, classifyFun and DTModels) provide confusion matrices for all the cross-validation schemes implemented when the parameter extendedResults is set to TRUE (*extendedResults = TRUE*). In cases where a k-fold or leave-one-subject-out cross-validation is utilized, the confusion matrices are summed together and subsequently the confusion matrix metrics are calculated (Forman & Scholz, 2010; Kelleher, Namee, & D’Arcy, 2015).

### Appendix References

Borra, S., & Di Ciaccio, A. (2010). Measuring the prediction error. A comparison of cross-validation, bootstrap and covariance penalty methods. *Computational Statistics & Data Analysis*, *54*(12), 2976–2989. 10.1016/j.csda.2010.03.004

Browne, M. W. (2000). Cross-Validation Methods. *Journal of Mathematical Psychology*, *44*(1), 108–132. 10.1006/jmps.1999.1279

Douglas, P. K., Harris, S., Yuille, A., & Cohen, M. S. (2011). Performance comparison of machine learning algorithms and number of independent components used in fMRI decoding of belief vs. disbelief. *NeuroImage*, *56*(2), 544–53. 10.1016/j.neuroimage.2010.11.002

Duda, R. O., Hart, P. E., & Stork, D. G. (2000). *Pattern Classification*. *Journal of Classification* (Vol. 24).

Forman, G., & Scholz, M. (2010). Apples-to-apples in cross-validation studies. *ACM SIGKDD Explorations Newsletter*, *12*(1), 49. 10.1145/1882471.1882479

Gong, G. (1986). Cross-Validation, the Jakknife, and the Bootstrap: Excess Error Estimation in Forward Logistic Regression. *Journal of the American Statistical Association*, *81*(393), 108–113. 10.1080/01621459.1986.10478245

Han, J., Kamber, M., & Pei, J. (2012). Classification. In *Data Mining* (pp. 327–391). Elsevier. 10.1016/B978-0-12-381479-1.00008-3

Kelleher, J. D., Namee, B. Mac, & D’Arcy, A. (2015). *Fundamentals of Machine Learning for Predictive Data Analytics*. Cambridge, Massachusetts: The MIT Press.

Kiang, M. Y. (2003). A comparative assessment of classification methods. *Decision Support Systems*, *35*(4), 441–454. 10.1016/S0167-9236(02)00110-0

Kim, J.-H. (2009). Estimating classification error rate: Repeated cross-validation, repeated hold-out and bootstrap. *Computational Statistics & Data Analysis*, *53*(11), 3735–3745. 10.1016/j.csda.2009.04.009

Kohavi, R. (1995). A Study of Cross-Validation and Bootstrap for Accuracy Estimation and Model Selection. *International Joint Conference on Articial Intelligence (IJCAI)* (pp. 1–7). 10.1067/mod.2000.109031

Song, X., Mitnitski, A., Cox, J., & Rockwood, K. (2004). Comparison of machine learning techniques with classical statistical models in predicting health outcomes. *Studies in Health Technology and Informatics*, *107*(Pt 1), 736–740.

Varoquaux, G., Raamana, P. R., Engemann, D. A., Hoyos-Idrobo, A., Schwartz, Y., & Thirion, B. (2017). Assessing and tuning brain decoders: Cross-validation, caveats, and guidelines. *NeuroImage*, *145*(Pt B), 166–179. 10.1016/j.neuroimage.2016.10.038

## Footnotes

1 An R data frame is an object used for storing data tables where each column is list of categorical or numeric data variables.

2 This method of parameter input is equivalent to a symbolic description of the to-be fitted model (e.g., classCol ~ feature1 + feature2, etc.).

3 Machine learning results can vary especially in small sample sizes or with disproportionate class sizes depending on the choice of cross-validation scheme. To reduce such effects, *PredPsych* utilizes a stratified cross-validation scheme and, by default, sets a fixed seed value (*SetSeed* = TRUE).

4 A mapping function that transforms input features into a higher dimensional space (Hofmann, Schölkopf, & Smola, 2008).
